# Syndromic Testing in Infectious Diseases: From Diagnostic Stewardship to Antimicrobial Stewardship

**DOI:** 10.3390/antibiotics12010006

**Published:** 2022-12-21

**Authors:** Oana Săndulescu, Anca Streinu-Cercel, Maria Magdalena Moțoi, Adrian Streinu-Cercel, Liliana Lucia Preoțescu

**Affiliations:** 1 Department of Infectious Diseases I, Faculty of Medicine, Carol Davila University of Medicine and Pharmacy, 050474 Bucharest, Romania; 2 National Institute for Infectious Diseases “Prof. Dr. Matei Balș”, 021105 Bucharest, Romania

**Keywords:** antimicrobial stewardship, antibiotics, viral infection, pneumonia, differential diagnosis

## Abstract

The implementation into clinical practice of syndromic testing by multiplex polymerase chain reaction allows early etiological diagnosis and paves the way towards timely targeted treatment. However, there is stringent need for diagnostic stewardship, as multiplex testing can also come with a high risk of misdiagnosis if improperly ordered or interpreted. We report two cases that illustrate proper and improper diagnostic stewardship, having important implications for correct patient management and application of antimicrobial stewardship into current clinical practice.

## 1. Introduction

The development and rapid uptake of multiplex polymerase chain reaction (PCR) tests into clinical practice has marked the beginning of a new era, that of early etiological diagnosis, paving the way towards timely targeted treatment. Syndromic-based testing opens up a world of possibilities and has the potential to improve clinical management and facilitate antimicrobial stewardship (AMS) if used correctly [1,2]. However, there is stringent need for diagnostic stewardship (DNS) to be put in place, as multiplex testing can also come with a high risk of misdiagnosis if improperly ordered or interpreted.

We hereby report two cases that are illustrative of proper and improper diagnostic stewardship, having important implications into correct patient management, and also of application of AMS principles into current clinical practice, to mark the World Health Organization’s World Antimicrobial Awareness Week.

## 2. Case Reports

### 2.1. Case Report 1

At the morning clinical rounds in the infectious diseases department, we were informed that a 60-year-old male patient had been admitted to the hospital on the previous night with a fever and productive cough. The patient was not in his hospital room that morning, because he was undergoing renal replacement therapy at a hemodialysis clinic and was scheduled to return back to our hospital by 11 a.m.

**Figure 1:**
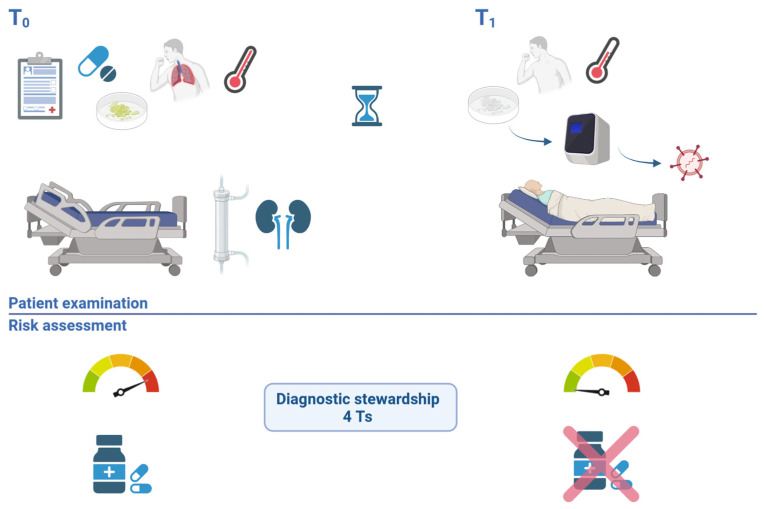
Case evaluation timeline–patient 1. T_0_ illustrates the initial risk assessment, in the absence of the patient, highlighting the high risk of a bacterial infection with a multidrug-resistant organisms in a patient with multiple comorbidities, immunosuppression, high fever, productive cough and recent antibiotic use. Diagnostic stewardship was applied (4 Ts): based on the right molecular Test, from the right Type of sample, collected at the right Time, Treatment was revised. T_1_ illustrates the new risk assessment performed after examining the patient, noting a good clinical state and ordering a molecular test that ruled out a bacterial infection, while confirming the viral etiology of pneumonia, allowing the stop of empirical wide spectrum antibiotherapy.

Consulting the patient’s medical record, we found the following information: he had been diagnosed with IgG kappa multiple myeloma 9 months previously, for which he had received one course of bortezomib + dexamethasone (Vd), two courses of daratumumab + bortezomib + thalidomide + dexamethasone (DVTd), followed by two courses of daratumumab + lenalidomide + dexamethasone (DRd) and an allogeneic bone marrow transplant completed 40 days prior to the current hospital admission. He also had a history of two episodes of pneumonia complicated with sepsis in between the chemotherapy sessions, the most recent one having been treated empirically with piperacillin-tazobactam, linezolid and fluconazole 5 months prior to the current hospital admission. He had presented to our hospital during the weekend with a two-day onset of fever (38.7 °C at the moment of admission), asthenia and a productive cough with self-described red-tinged sputum (Figure 1 T_0_).

Other comorbidities included therapeutically controlled hypertension, controlled type II diabetes mellitus, partially corrected severe pancytopenia following bone marrow transplant, hypertensive cardiopathy, depressive syndrome with one prior episode of suicide attempt and end-stage kidney disease for which he was undergoing hemodialysis three times per week on a central venous catheter.

The laboratory workup at hospital admission revealed the following: normal white blood cell count, severe chronic anemia, mild thrombocytopenia and normal serum biochemistry. The C-reactive protein result was pending. 

**Figure 2:**
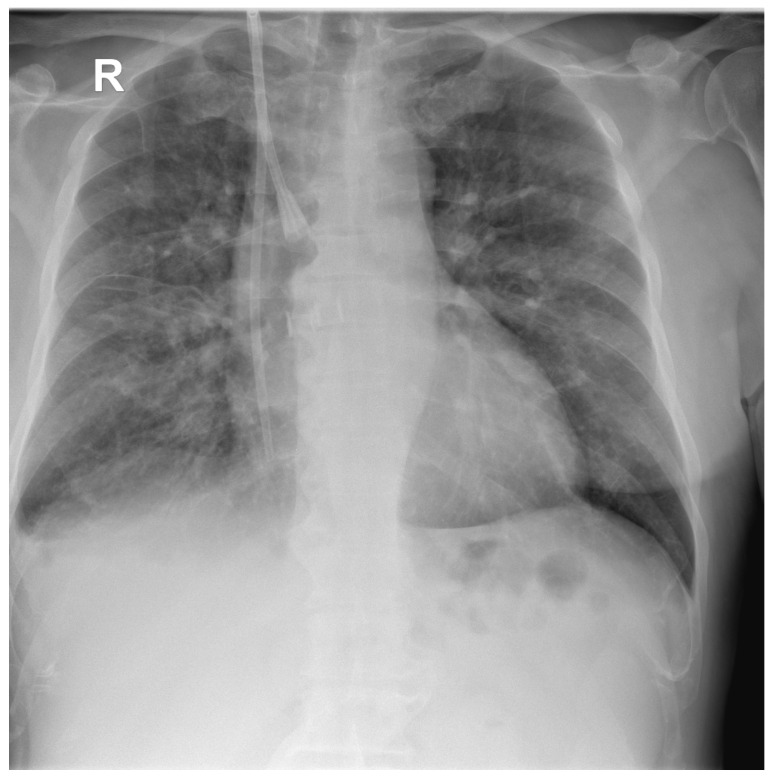
Postero-anterior chest X-ray of the first patient at hospital admission showing multiple hazy areas of mixed (alveolo-interstitial) infiltrates scattered throughout both lungs, and central venous catheter with insertion in the right internal jugular vein and internal end in the right atrium.

A chest X-ray (Figure 2) at admission showed moderate–severe mixed lung infiltrates, predominantly over the right lung field, on a fibrous background with minimal right pleural reaction.

The patient was considered to be at high risk for a bacterial infection; therefore, empirical antimicrobial therapy was ordered, to cover the most likely Gram-negative pathogens with meropenem, adjusted for hemodialysis to 500 mg Q24h and a potential catheter infection with methicillin-resistant *Staphylococcus aureus* (MRSA), with teicoplanin at a loading dose of 600 mg Q12h for three doses, to be followed by 600 mg Q72h.

After the patient returned from hemodialysis, we were able to perform the full clinical consult. The patient was afebrile at the time of evaluation, and was apparently feeling well. He was obese (body mass index of 32 kg/m^2^) and had normal breath sounds, plus rare wet crackles basally in both lungs, with occasional productive cough but non-purulent sputum. The rest of the clinical exam was non-suggestive. The anamnesis revealed an epidemiological contact within the previous week with a 3-year old nephew who had “a cold”.

Given the patient’s overall good clinical state, the absence of fever and the epidemiological contact, a multiplex respiratory panel was considered. Because the patient also presented productive cough, a sputum sample was requested and was obtained later within the evening of the same day, when it was also sent to the hospital’s microbiology laboratory. Giemsa- and Gram- colored smears identified > 25/100x inflammatory cells, of which 40% polymorphonuclear cells and 60% mononuclear cells, fibrin (3+), and rare extraleukocyte Gram-positive cocci in small clusters. A lower respiratory tract panel (FilmArray Pneumonia (PN) Panel, BioFire Diagnostics LLC, Salt Lake City, UT, USA) was performed from sputum. It ruled out the presence of the following bacterial pathogens: *Acinetobacter calcoaceticus-baumannii* complex, *Enterobacter cloacae* complex, *Escherichia coli, Haemophilus influenzae*, *Klebsiella aerogenes*, *Klebsiella oxytoca*, *Klebsiella pneumoniae* group, *Moraxella catarrhalis*, *Proteus* spp., *Pseudomonas aeruginosa*, *Serratia marcescens*, *Staphylococcus aureus*, *Streptococcus agalactiae*, *Streptococcus pneumoniae*, *Streptococcus pyogenes*, *Chlamydia pneumoniae*, *Legionella pneumophila*, *Mycoplasma pneumoniae*, the following antimicrobial resistance genes: carbapenemases (IMP, KPC, NDM, OXA-48-like, VIM), ESBL (CTX-M), methicillin resistance (*mecA/C*, MREJ), and the following viral pathogens: adenovirus, human coronavirus, human metapneumovirus, human rhinovirus/enterovirus, influenza A virus, influenza B virus and respiratory syncytial virus. The same panel identified parainfluenza virus as the etiological agent of the patient’s infection.

At this moment, the therapeutic regimen was immediately reconsidered (Figure 1, T_1_). Blood cultures drawn at hospital admission and drawn from the hemodialysis catheter the previous day were not presenting any signs of bacterial growth yet, and neither did the sputum cultures. Therefore, after having confirmed the viral etiology of the infection, both antimicrobials started empirically the previous day were stopped. The patient remained hospitalized for 3 more days, during which he remained afebrile; the cough became dry and unproductive, and the breath sounds remained normal with a decreasing intensity of the wet crackles, paralleled by a marked decrease in C-reactive protein (Table 1). He was discharged after a total hospital stay of 5 days, afebrile, with good general condition and normal lung clinical exam, with the recommendation to continue monitoring his temperature and the intensity and nature of the residual cough. Incubation of blood cultures continued post-discharge to complete a total duration of 7 days, until returning a final negative result. At one week follow-up post discharge, the patient had no signs of acute infection, a residual dry cough and normal pulmonary auscultation.

### 2.2. Case Report 2

A 65-year-old female patient with therapeutically controlled hypertension, ischemic heart disease and overweight status presented to the hospital’s outpatient department for a two-day onset of fever, malaise, dry cough and nasal congestion. She tested negative for SARS-CoV-2 and influenza antigen, and was tested by multiplex PCR from a nasopharyngeal swab with an upper respiratory tract panel (FilmArray Respiratory 2.1 plus Panel, BioFire Diagnostics LLC, Salt Lake City, UT, USA), which detected human rhinovirus/enterovirus. Clinical exam was normal at this time point, with no findings on lung auscultation. No further laboratory tests were performed at this point, and she was treated on an outpatient basis with non-steroidal anti-inflammatory drugs (NSAIDs).

Four days later, she returned for evaluation for persistence of malaise, dry cough and nasal congestion in the absence of fever. She had performed a new multiplex PCR test in an external clinic, this time a lower tract respiratory panel (FilmArray Pneumonia (PN) Panel, BioFire Diagnostics LLC, Salt Lake City, UT, USA). However, since the patient’s cough was non-productive and a bronchoalveolar lavage was not indicated, no suitable sample had been obtained, and a nasopharyngeal swab was used instead. The test detected human rhinovirus/enterovirus plus *Haemophilus influenzae* 10^4^ copies/mL and *Moraxella catarrhalis* 10^4^ copies/mL. Based on these results, the patient returned to our clinic with the suspicion of bacterial superinfection pneumonia.

**Figure 3:**
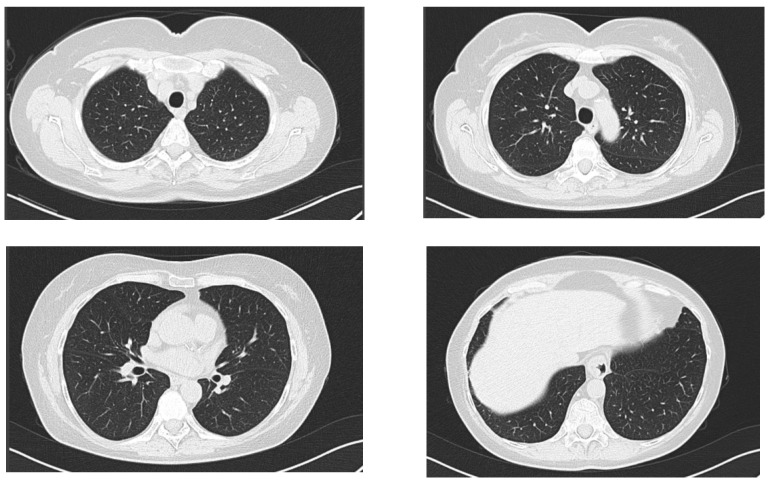
Axial computed chest CT of the second patient, lung window at the following levels: supraaortic trunks, aortic arch, pulmonary hila and base of the lungs, without any evidence of acute pulmonary lesions.

Clinical exam at this time point was mostly non-remarkable, with slightly coarse breath sounds without crackles. A native chest CT was performed, and it ruled out an acute pneumonia (Figure 3). A complete blood count was normal (8900 white blood cells/µL, of which 5600 neutrophils/µL and 2200 lymphocytes/µL, 12.2 g/dL hemoglobin and 259,000 platelets/µL. Fibrinogen was normal (275 mg/dL, normal range: 200–393 mg/dL), erythrocyte sedimentation rate was normal (32 mm/h normal range: [age (years) + 10]/2), mild liver cytolysis (ALT 98 U/L, normal range: 4–35 U/L; AST 77 U/L, normal range: 14–36 U/L), and dyslipidemia (total cholesterol 244 mg/dL, normal range: 50–200 mg/dL; triglycerides 196 mg/dL, normal range: 15–150 mg/dL).

A diagnosis of superinfection pneumonia was ruled out based on the absence of suggestive clinical findings (the patient presented neither of: productive cough, thoracal pain, dyspnea, fever), fairly normal lung auscultation, normal lung imaging and normal laboratory tests (there was no leukocytosis, no neutrophilia and no inflammatory syndrome). The bacterial agents identified in the second respiratory panel were interpreted as colonizers of the upper airways. An antibiotic was not indicated in this case, and the patient continued to be monitored as outpatient, with NSAIDs alone, and with full clinical recovery over the next 3 days and a normal clinical exam at a two-week follow-up. 

## 3. Discussion

Here, we have presented two cases that are illustrative of the importance of implementing diagnostic stewardship. In the first case, the availability of a multiplex PCR respiratory panel allowed the stopping of empiric wide-spectrum antibiotic treatment and an early discharge from the hospital. In the second case, however, a multiplex PCR respiratory panel was performed from an inadequate sample type, in the absence of diagnostic stewardship, which lead to an inappropriate interpretation of the detected organisms as pathogens, even though these were in fact low gene counts of two upper airway colonizers. 

This had the potential to lead to an incorrect diagnosis of bacterial superinfection pneumonia and the administration of unnecessary antibiotics. Fortunately, the principles of rational antimicrobial prescription were applied here, and a comprehensive evaluation of the case excluded the diagnosis of bacterial pneumonia and confirmed that this was actually the natural course of the patient’s viral infection. 

With increased uptake of multiplex panels for syndromic testing in infectious diseases, a growing body of knowledge and experience has accumulated. The clinical significance of the semi-quantitative gene count results remains, however, an area that warrants further research. These values should be interpreted with caution, even for correctly performed tests, as there currently is no clear clinical validation for translating this gene target quantification into a definite differentiation between infection vs. colonization. Recent studies have shown that multiplex panel gene counts in the range of 10^4^–10^5^ copies/mL will not be culturable in 63.5% of cases [3], and that higher panel thresholds, such as 10^6^ and above for *Staphylococcus aureus* and ≥10^7^ for *Haemophilus influenzae* have the best likelihood of being associated with bacterial growth in concomitant standard cultures [4]. For bronchoalveolar lavage, bacterial culture colony forming units (CFU) and multiplex panel genomic copies count are reported to be concordant in 47.4% of cases [5]; concordance of these gene counts with CFU values was seen for *S. aureus* starting from 10^5^ gene target counts and for *Klebsiella aerogenes* from ≥10^7^ gene target counts [6].

A concomitant strength and limitation of PCR tests in general is that they identify genetic material without differentiating between viable or non-viable organisms. In situations where empirical antimicrobial treatment has already been initiated prior to sample collection, this could provide to be advantageous, allowing an etiological diagnosis to be established where standard culture would have failed. However, this also carries the risk of overinterpreting bacterial prints as pathogens when they rightly may not be. Therefore, scrupulous clinical judgement should be applied when interpreting the results of multiplex panels, as with any microbiological test result.

Ideally, in healthcare settings, diagnostic stewardship and antimicrobial stewardship should both be consistently applied [7] in order to ensure that the correct diagnostic test is performed, and that the results will inform clinical practice on whether or not the prescription of an antibiotic treatment is warranted. While antimicrobial stewardship is being increasingly implemented in healthcare facilities, the principles of diagnostic stewardship are only now starting to be established. To implement DNS, the importance of correct specimen type and the interpretation of results should be communicated from the laboratory to clinicians. This could potentially be done through rejection of wrong specimen types from the laboratory, along with a standardized explanation of the reasons behind this decision, communicated either directly or through digital nudging and coupled with provider education initiatives. Furthermore, a dedicated DNS team could review requests for multiplex PCR tests to ensure adequacy and application of DNS principles, while the AMS team could review the results of multiplex PCR tests to ensure correct interpretation and correct subsequent treatment decisions.

When a patient presents with fever, we first need to establish: is the fever due to an infection? This is particularly important, as several hematological, neoplastic or autoimmune diseases can also present with fever and be initially misdiagnosed as infection [8,9]. If there is a good reason to consider that fever is indeed due to an infection, particularly if the patient is severely ill or has signs suggestive for a primary site of infection, i.e., urinary, pulmonary, etc., the clinician has to further perform a differential diagnosis to establish whether the etiology is most likely viral or bacterial [10]. Additionally, if there is a high suspicion of bacterial infection, only then does the process of choosing the right antibiotic start [11].

**Figure 4:**
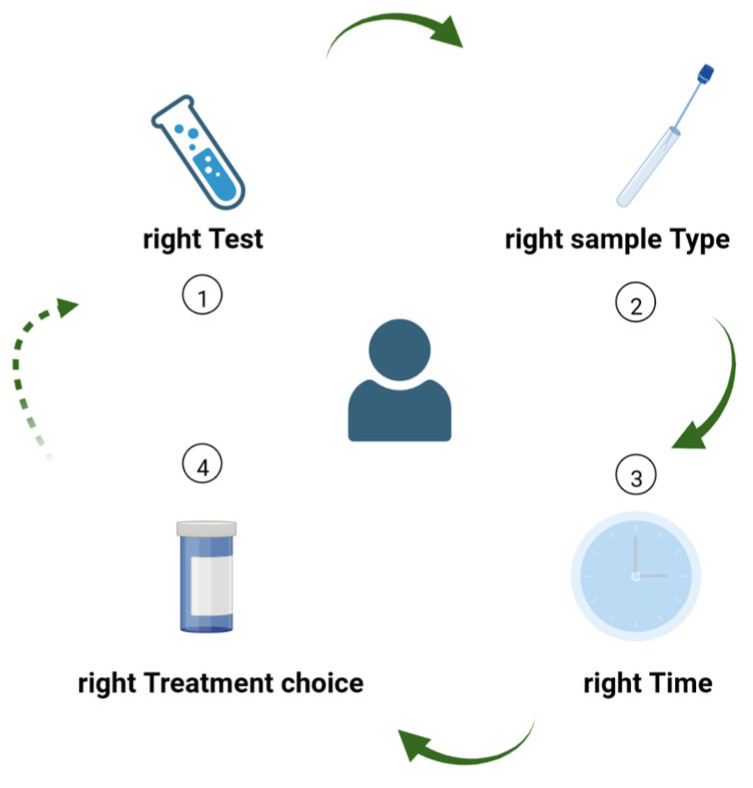
The 4 Ts of diagnostic stewardship: (1) choosing the right Test from (2) the right Type of sample, collected at (3) the right Time, in order to (4) guide Treatment decisions. The process can be restarted whenever a new laboratory test has the potential to further guide the diagnostic or therapeutic process, or to better monitor the patient’s evolution under treatment.

Diagnostic stewardship is essential to guide clinical practice and to enable AMS. Much like the 4 Ds of optimal antimicrobial therapy [12], which are essential components of AMS programs, we can rethink a set of 4 Ts for diagnostic stewardship (Figure 4), specifically: choosing the right Test from the right Type of sample, collected at the right Time, in order to guide Treatment.

## 4. Conclusions

We have highlighted how novel diagnostic methods can either aid or hinder clinical practice, based on the appropriateness of their use. Furthermore, consistent implementation of the 4 Ts of diagnostic stewardship can enable better antimicrobial stewardship, particularly in specific patient populations that would otherwise be considered to be at high risk of multidrug-resistant bacterial infections.

## Figures and Tables

**Table 1:** Laboratory workup for Case 1.

Title 1	Day 1	Day 3	Day 5
White blood cell count (normal range: 3600–9600 cells/µL)	7600	**3300**	4100
Neutrophil count (normal range: 1400–6500 cells /µL)	5200	1800	1700
Lymphocyte count (normal range: 1200–3400 cells/µL)	1800	**1100**	1700
Hemoglobin (normal range: 12.1–17.2 g/dL)	**7.5**	**7.9**	**8.1**
Platelet count (normal range: 200,000–400,000 cells/µL)	**122,000**	**118,000**	**138,000**
Fibrinogen (normal range: 200–393 mg/dL)	379	372	386
C-reactive protein (normal range: (0–3 mg/L)	**82.6**	**21.5**	-

Values outside of the laboratory’s normal range are indicated by bold font.

## Data Availability

Not applicable.
